# Colonization by *Panstrongylus megistus* (Hemiptera, Reduviidae, Triatominae) in an urban park in the city of São Paulo

**DOI:** 10.1590/0037-8682-0330-2020

**Published:** 2020-12-21

**Authors:** Rubens Antonio da Silva, Vera Aparecida Oliveira Estevão, Agnaldo Nepomuceno Duarte, Priscilla Cipolini Maria

**Affiliations:** 1 Superintendência de Controle de Endemias, Departamento de Combate a Vetores, Laboratório Especializado de Mogi Guaçu: Doença de Chagas, São Paulo, SP, Brasil.; 2 Superintendência de Controle de Endemias, Centro Regional Região Metropolitana de São Paulo, SP, Brasil.; 3 Secretaria de Infraestrutura e Meio Ambiente, Parque Estadual Dr. Fernando Costa Água Branca, São Paulo, SP, Brasil.

**Keywords:** São Paulo, Chagas disease, Triatomines, Urban parks

## Abstract

**INTRODUCTION:**

This communication reports on the occurrence of colonization by *Panstrongylus megistus in an urban park in the municipality of São Paulo*, Brazil.

**METHODS:**

Entomological research includes active search for vectors based on notifications by the population and identification and examination of insects.

**RESULTS:**

A colony of triatomines was found to be associated with enclosed birds.

**CONCLUSIONS:**

The occurrence of *P. megistus* has already been reported in the city of São Paulo; however, reports of colonization by this species provide evidence of its potential for the occupation of artificial ecotopes, which may pose a risk to the human population.

Triatomines are insects belonging to the subfamily Triatominae (Hemiptera: Reduviidae). They are responsible for the transmission of *Trypanosoma cruzi*, the etiological agent of Chagas disease, to humans, and to a large variety of wild and domestic animals. A total of 154 triatomine species have been reported^1^, all of which are potential vectors of *T. cruzi*, presenting extensive morphological variation and different vector capacities. The majority of triatomine species maintain strictly wild habits, while others have adapted to life in anthropic environments^2^.

Among the forms of transmission, the oral one, which used to be considered sporadic in humans, has been occurring somewhat frequently^3^. Oral outbreaks are responsible for the growing number of new cases of the acute form of the disease and for the increased morbidity and mortality. Such outbreaks have been related to the consumption of contaminated food as well as the invasion of wild habitats by humans, which increases the risks associated with proximity to vectors and reservoirs^4^.

The state of São Paulo was once considered to have one of the highest prevalences of Chagas disease in the country, with *Triatoma infestans* being widely distributed. São Paulo was also a pioneering area for the development of a regular vector transmission control program, which started in the 1950s and was still successful in the 1970s, thus serving as a model for the creation of the Brazilian National Program. The program, together with the experiments carried out in Bambuí - MG, became the root of other programs in South America^5^. A total of 14 triatomine species have been reported so far, with the most collected species being *Triatoma sordida* (Stal, 1839), *Panstrongylus megistus* (Burmeister, 1835), *Rhodnius neglectus* (Lent, 1954), and *T. tibiamaculata* (Pinto, 1926). Due to its presence in the intra - and peri-domicile of human habitations and to the high rates of natural infection, *P. megistus* has been considered the species with the highest epidemiological importance in the state^5^. 

In the city of São Paulo, sporadic findings of triatomine bugs have been reported since the 1990s, without colonization, and *P. megistus* is the species with the highest number of records^6^. The first colonization of this species in the Metropolitan Region of São Paulo occurred in the municipality of Carapicuíba in 2018, in a house located in a condominium surrounded by vegetation^7^.

The finding of triatomines in urban parks, which are considered green areas with an ecological, esthetic, and leisure function and which have large extensions than that of squares and public gardens, has never been reported in the state. The objective of our study was to report, for the first time, the occurrence of a colony of *P. megistus* in an urban park located in the municipality of São Paulo.

The city of São Paulo has 101 urban parks, which are managed by municipal or state governments. Located in the Água Branca district, close to the Barra Funda and Perdizes districts, *Parque Dr. Fernando Costa - Água Branca* is an urban park used by a diversified public for hiking, running, physical activities, meditation, and visiting different farmer associations present in the park. On weekends, people visit the park for leisure and to attend exhibitions, events, and enjoy the food kiosks (Figure 1). In the park, there are residual forests, areas with eucalyptus, open spaces, and marshy areas. It shelters more than a thousand gallinaceous birds as well as ducks, peacocks, and swans. 

**FIGURE 1:**
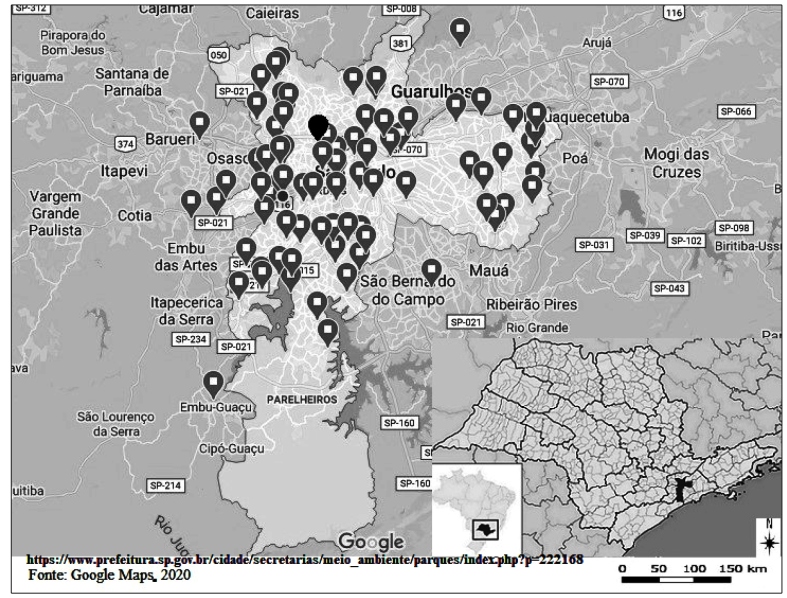
Urban parks managed by the municipal or state governments. In black: *Parque Dr Fernando Costa - Água Branca*. Municipality of São Paulo, 2020.

Entomological surveillance of Chagas disease in São Paulo is passive and activated when a citizen sends a triatomine bug to one of the city’s notification points for triatomines. The research includes an active search for triatomines by the field teams of Sucen (Superintendence for Endemics Control) or of the municipality, in the home unit, intra - and peri-domicile, with special attention to places that shelter animals and function as food sources for triatomines. When a vector is detected, the insects are collected, placed in plastic containers, labeled, and sent to the entomology laboratory for identification and examination of intestinal contents for verification of positivity for trypanosomatids. Also, the intestinal content is used to check eating habits against antisera, human, marsupial, rodent, bird, dog and cat, using the ELISA technique^8^. The area where the presence of a triatomine is detected is subjected to mechanical and chemical control with the utilization of insecticides distributed by the Brazilian Ministry of Health.

Since 2016, the metropolitan region of São Paulo, which includes 39 municipalities, has suffered pressure from the *P. megistus* species, with frequent invasions in domiciles located in the urban area, mostly in condominiums. This species has used an ecological corridor formed by forest fragments in urban areas, mostly represented by parks, and from the corridor, it has invaded domiciles. From 2016 to 2020, 115 notifications of this species were received, and 196 triatomine specimens in nine different municipalities were collected (Table 1).

**TABLE 1:** Triatomines of the species *Panstrongylus megistus* captured, examined, and positive in the notification by residents and in attendance with the notification. Metropolitan Region of São Paulo, 2016 to 2020*.

Year	Municipality	Notification			Attendance to notification		
		Captured	Examined	Positive	Captured	Examined	Positive
2016	Pirapora do Bom Jesus	1	0	0	0	0	0
	São Bernardo do Campo	1	0	0	0	0	0
	Taboão da Serra	7	7	2	0	0	0
Subtotal		9	7	2	0	0	0
2017	São Paulo	3	0	0	0	0	0
	Taboão da Serra	25	25	6	0	0	0
Subtotal		28	25	6	0	0	0
2018	Carapicuíba	4	4	0	15	15	0
	Embu das Artes	2	2	2	0	0	0
	Itapecerica da Serra	3	3	0	0	0	0
	São Paulo	2	2	0	0	0	0
	Taboão da Serra	24	23	17	0	0	0
Subtotal		35	34	19	15	15	0
2019	Carapicuíba	2	2	0	38	11	0
	Cotia	2	2	0	0	0	0
	Embu das Artes	1	1	1	0	0	0
	Santana de Parnaiba	1	1	0	0	0	0
	São Paulo	9	5	1	12	4	0
	Taboão da Serra	22	22	12	12	12	0
Subtotal		37	33	14	62	27	0
2020	São Paulo	5	5	0	0	0	0
	Taboão da Serra	5	5	3	0	0	0
Subtotal		10	10	3	0	0	0
**Total**		**119**	**109**	**44**	**77**	**42**	**0**

*Up 04/20/2020.

There is a large number of gallinaceous birds of different species, both in enclosures and moving freely, in the Água Branca Park. Based on the movement displayed by triatomines in the Metropolitan Region of São Paulo, and more specifically in the municipality of São Paulo, an entomological survey was carried out on the park's premises by the field teams of the Sucen in October 2019. Survey of animal’s shelters of different species, bird nests, tree trunks, inlays, and tree hollows led to the collection of 12 specimens of *P. megistus* triatomines: one adult female specimen, one 2^nd^ stage nymph, four 3^rd^ stage nymphs, three 4^th^ stage nymphs, and three 5^th^ stage nymphs. These vectors were collected from the enclosure of gallinaceous birds, and four specimens (two 3^rd^ stage nymphs, one 4^th^ stage nymph, and one 5^th^ stage nymph) were examined and tested negative for trypanosomatid infection. Examination of the eating habits of the four specimens revealed the presence of bird blood. Chemical control was performed using Alphacypermethrin insecticide in the birds’ enclosure. Subsequent entomological research carried out four months after the discovery of the first colony did not detect new specimens.

In the municipality of São Paulo, forest fragments can be found in parks, squares, and environmental protection areas. After the control of *T. infestans*, *P. megistus* is considered the main vector of *T. cruzi* in Brazil. In addition to its wide geographical distribution, it presents high infection rates by *T. cruzi*, great anthropophilia, and a remarkable capacity to colonize artificial ecotopes, establishing peri and intradomiciliary colonies in some cases. The adaptation of this native species to the domiciliary environment is directly related to the action of humans on the environment and the reduction of its habitual food sources^9^.

In the state of São Paulo, *P. megistus* has a restricted distribution range. Its survival is favored by the rainfall regime, greater humidity, and type of vegetation cover, and is associated with marsupials of the Didelphidae family and rodents, resulting in a high rate of natural infection^11^ ^,^ ^11^. This species can colonize the human environment and maintain the circulation of *T. cruzi* in the area.

In line with the observations that *P. megistus* species is a highly dispersive agent in the Brazilian territory, with recognized potential for infestation and colonization of domiciles and high infection levels, increased detection and infection rates have been reported over the years. The absence of *T. cruzi* infection in the colony reported in this study can be related to the fact that the insects were associated with bird blood, and birds are refractory to the parasite infection. It should be mentioned here that findings of triatomines with *T. cruzi* infection invading domiciles have been previously reported in the municipality of São Paulo^12^. 

In Salvador, the state of Bahia, Dias Lima, and Sherlock^13^ reported the presence of adult specimens of the *T. tibiamaculata* species invading condominiums located in an urban area with high natural infection rates. In Corrientes, Argentina, the presence of *T. sordida* was reported in dwellings located in an urban area, but no infection by *T. cruzi* was detected^14^.

The prevalence of nymphs among the specimens captured in the municipality of São Paulo is characteristic of the capacity of adaptation of triatomines to the artificial ecotope, consolidated in the domiciliation process. This finding, which is in line with the movement presented by this species of triatomine in the municipality and also with the large supply of food, mainly represented by birds in the researched environment, highlights its importance for the municipality.

In this context, notifications from the public about suspicious insects are an important tool which must be implemented and enhanced in the entire region. Improvement in surveillance systems depends on the correct identification of vectors. 

Deforestation, fires, and alterations to the natural environment have become increasingly frequent. This, together with current changes in climate and human behavior patterns, can change the cycle of transmission of certain diseases, which can cause the reemergence of illnesses that had been considered controlled.

The Metropolitan Region of São Paulo has been affected by the presence and colonization of triatomine bugs in urban areas, with an increasing number of notifications by its municipalities on an annual basis. Dogs, humans, birds, and opossums have served as food sources for these triatomines. The occurrences, which used to be sporadic at the beginning of the 2010s^6^, are nowadays frequent in municipalities of this region. Further, municipalities which had never had reports of triatomines are now reporting them^15^. 

In view of this, measures related to the control of triatomines, such as active search for vectors, environmental management with modification of conditions that allow the presence and maintenance of these vectors and chemical control, must be used. Careful and well conducted research, together with appropriate chemical control and constant participation of the population in the notification of suspected insects have been the mechanisms that have guaranteed sustainability^5^. When a colony of kissing bugs is eliminated, should the same conditions persist, a new colonization can occur within a period of four to six months^9^. In this context, differences in the life cycle of the species, the availability of a food source, and the instability of the action of insecticides in the home environment, among other factors, should be taken into account. 

There is currently a large number of gallinaceous birds in the Água Branca Park, whether in confinement or moving freely in the area. Measures based on the presence of gallinaceous birds must be implemented. In addition, and considering that the species *P. megistus* has a dispersion radius of 400 m^9^, educational efforts targeted at the population living in the surroundings of urban parks should be considered as an alternative measure for the monitoring of Chagas disease vectors in the municipality of São Paulo. Furthermore, given that the population in urban areas has not experienced the transmission of Chagas disease, they may be unaware of the vector and need to be educated on the places where they can send suspicious insects.

## ETHICAL CONSIDERATIONS

The collection of triatomines follows the protocols established by São Paulo’s State Department of Health, which are compliant with the Brazilian Ministry of Health.
